# The experience of blood glucose monitoring in people with type 2 diabetes mellitus (T2DM)

**DOI:** 10.1002/edm2.302

**Published:** 2021-12-17

**Authors:** Mike Stedman, Rustam Rea, Christopher J. Duff, Mark Livingston, Katie McLoughlin, Louise Wong, Stephen Brown, Katherine Grady, Roger Gadsby, John M. Gibson, Angela Paisley, Anthony A. Fryer, Adrian H. Heald

**Affiliations:** ^1^ Res Consortium Andover UK; ^2^ Oxford Centre for Diabetes, Endocrinology and Metabolism Oxford UK; ^3^ Department of Clinical Biochemistry North Midlands and Cheshire Pathology Service University Hospitals of North Midlands NHS Trust Stoke on Trent UK; ^4^ School of Medicine Keele University Keele UK; ^5^ Black Country Pathology Services Walsall Manor Hospital Walsall UK; ^6^ Salford Royal Hospital Salford UK; ^7^ Warwick Medical School University of Warwick West Midlands UK; ^8^ The School of Medicine and Manchester Academic Health Sciences Centre University of Manchester Manchester UK

**Keywords:** blood glucose, diabetes education, HbA1c, monitoring, patient experience, type 2 diabetes

## Abstract

**Background:**

Finger prick blood glucose (BG) monitoring remains a mainstay of management in people with type 2 diabetes (T2DM) who take sulphonylurea (SU) drugs or insulin.

We recently examined patient experience of BG monitoring in people with type 1 diabetes (T1DM). There has not been any recent comprehensive assessment of the performance of BG monitoring strips or the patient experience of BG strips in people with T2DM in the UK.

**Methods:**

An online self‐reported questionnaire containing 44 questions, prepared following consultation with clinicians and patients, was circulated to people with T2DM. 186 responders provided completed responses (25.5% return rate). Fixed responses were coded numerically (eg not confident = 0 fairly confident = 1).

**Results:**

Of responders, 84% were treated with insulin in addition to other agents. 75% reported having had an HbA1c check in the previous 6 months.

For those with reported HbA1c ≥ 65 mmol/mol, a majority of people (70%) were concerned or really concerned about the shorter term consequences of running a high HbA1c This contrasted with those who did not know their recent HbA1c, of whom only 33% were concerned/really concerned and those with HbA1c <65 mmol/mol of whom 35% were concerned.

Regarding BG monitoring/insulin adjustment, only 25% of responders reported having sufficient information with 13% believing that the accuracy and precision of their BG metre was being independently checked. Only 9% recalled discussing BG metre accuracy when their latest metre was provided and only 7% were aware of the International Standardisation Organisation (ISO) standards for BG metres. 77% did not recall discussing BG metre performance with a healthcare professional.

**Conclusion:**

The group surveyed comprised engaged people with T2DM but even within this group there was significant variation in (a) awareness of shorter term risks, (b) confidence in their ability to implement appropriate insulin dosage (c) awareness of the limitations of BG monitoring technology. There is clearly an area where changes in education/support would benefit many.

## Graphical abstract

We conducted an online survey of experience of BG monitoring in people with T2DM. Perceptions of shorter vs longer term consequences of hyperglycaemia varied widely. Only 9% recalled discussing BG metre accuracy when their latest metre was provided and 77% did not recall discussing BG metre performance with a healthcare professional ‐ this is clearly an area where changes in education and support would benefit many.

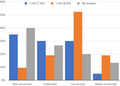

## 1 INTRODUCTION

In the last 40 years, self‐monitoring blood glucose (SMBG) has revolutionized the treatment of diabetes mellitus (DM). BG monitoring remains a mainstay of management in people with type 2 diabetes (T2DM) who take sulphonylurea (SU) drugs or insulin. However it has also been found to improve outcomes in people who are not taking these agents.^1^, ^2^

Although self‐monitoring of blood glucose is now widely accepted as part of the management of people with type 2 diabetes,^3^ Polonsky et al^4^ showed that lack of understanding and limited skills to apply self‐monitoring data to aid insulin dose adjustment, avoidance of thinking about BG values and diabetes, and a sense of pointlessness of self‐monitoring were associated with infrequent self‐monitoring and limited use of self‐monitoring data for insulin dose adjustments.

At a general practice level using National Diabetes Audit data, we have found that there is a significant variation in BG metre strip precision, which is linked to a variation in measured BG and in glycated haemoglobin (HbA1c).^5^ We recommended that account be taken of International Organisation for Standardisation (ISO) BG strip performance standards when BG metres are provided.^6^

Furthermore, we previously showed a link between analytical BG metre precision and an established qualitative error grid, highlighting the potential impact of accuracy on clinical decision and outcomes.^7^ Specifically, those metres with a variability of readings between 10% and 20% versus the standard laboratory method fall into the category of potentially affecting clinical outcomes for patients. This difference in BG monitor strip performance could mean that people make potentially harmful decisions about their food intake and insulin dose, based on imprecise BG readings.

We recently examined patient experience of BG monitoring in type 1 diabetes (T1DM) and drew some important conclusions.^8^ There has not been any recent comprehensive assessment of the performance of BG monitoring strips or the patient experience of BG strips in T2DM in the UK. Our study aimed to start to address this by asking people with T2DM about their experience of day‐to‐day BG monitoring and how this influenced their decisions about insulin dosing. Furthermore, confidence in the BG monitoring equipment is essential, so how patients felt and behaved in this critical area was also examined.

## 2 METHODS

A digital questionnaire containing 44 questions (See Appendix 1) was prepared in consultation with clinicians and patients and was sent by email to patients on the Research for the Future (RftF) consent for approach database.^9^ This is a National Institute for Health Research (NIHR) Clinical Research Network Greater Manchester initiative to encourage people with diabetes and other long‐term conditions living in the region to be more involved with local NHS health research opportunities.

Research for the Future approached their volunteers with T2DM by email inviting them to participate in the survey. This included a link to an online participant information sheet (PIS), consent and questionnaire. This online survey was conducted with the support of RftF and the sponsorship of Salford Royal Foundation Trust. Ethics approval was obtained prior to the survey being sent out.

Responses from the survey were allocated specific numerical values (eg not confident = 0, fairly confident = 1, and so on) on a Likert Scale and the responses to certain questions were related to self‐reported HbA1c.

Ethical approval was obtained from the West Midlands Research Ethics Committee: REC Reference: 19/WM/0075.

### 2.1 Statistics

Categorical responses were shown as simple percentages by levels and qualitative responses aggregated under headings. These are shown in the Figures.

## 3 RESULTS

### 3.1 Demographics & description of study population

In relation to the online survey, 186/730 (25.5%) of those individuals approached to complete the online questionnaire responded. 84% were treated with insulin in addition to other agents. 23% of respondents were 60 years old or younger and 72% had been diagnosed with T2DM for more than 10 years. Of all respondents, 37% were women. The non‐responder rate was 62%.

The characteristics of non‐responders were not materially different with 39% being women and 26% 60 years old or younger.

Of the respondents on insulin, 48% were injecting rapid acting insulin and 39% of insulin treated respondents said that they adjusted the dose of insulin that they administered. 24% reported giving insulin 4 or more times a day. 30% reported a daily insulin dose of up to 30 units per day, 35% between 31 and 60 units per day and 32% gave more than 60 units per day, with 3% unsure.

When asked about glycaemic control (4% of those questioned did not reply to this question), 51% of patients self‐reported their last HbA1c result as ≤64 mmol/mol (≤8.0%) and 7% reported their last HbA1c to be >86 mmol/mol (>10.0%). 75% reported having had an HbA1c check in the previous 6 months.

### 3.2 BG metre use

In relation to frequency of monitoring, 13% were testing once a day, 32% twice a day, 27% three times a day, 12% four times a day and 9% more than four times per day with 7% not at all. 63% stated that they were confident in the accuracy of their metres.

Regarding duration of BG metre use, 67% said that they had used a BG metre for more than 10 years. 51% had used the same BG metre for 3 years or more. 44% reported having been trained to use their BG metre. Concerning the matter of keeping a BG diary, 42% reported keeping a diary of BG readings consistently, 25% sometimes and 33% not at all.

In regards to difference between 2 consecutive readings when assessing metre accuracy, 18% of people reported a difference of more than 1.0 mmol/L, while 24% reported having to adjust their dose of insulin after a double check of the blood glucose reading.

**FIGURE 1:**
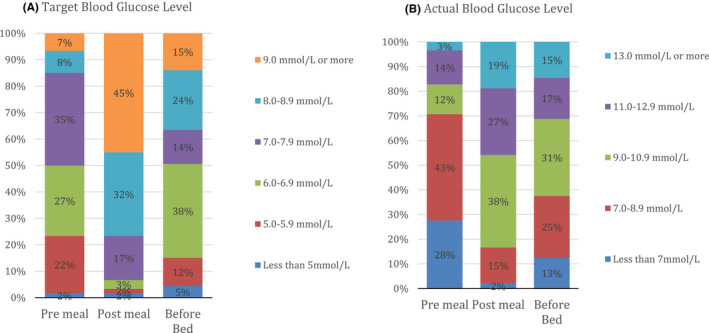
(A) Target blood glucose (BG) Level (mmol/L). (B) Actual blood glucose (BG) Level (mmol/L)

The distribution of reported BG target (Figure 1A) and actual readings (Figure 1B) are described. 50% were setting a target for pre‐meal readings ≥7 mmol/L with 45% setting a post‐meal target of ≥9 mmol/L. This relates to the concerns that respondents reported in relation to the consideration of potential hypoglycaemia as reported below. The actual recorded %BG readings pre‐meal ≥7 mmol/L was 70% with the actual recorded post‐meal ≥9 mmol/L being 67% of BG readings. For bedtime readings 15% were set at ≥9 mmol/L and for actual bedtime readings 50% were ≥9 mmol/L.

### 3.3 Respondent concerns and confidence

We next asked questions around how people with T2DM felt about BG levels and insulin dosing, 30% stated that they keep BG level high at times, to avoid hypoglycaemic episodes; this was reflected in the targets they set for pre‐meal and post‐meal BG levels. Furthermore, 52% were concerned that they might be over‐ or under‐dosing their insulin (Breakdown by HbA1c is shown in the Figure S1, which illustrates that this proportion increased to 66% in those whose last reported HbA1c was ≥65 mmol/mol).

**FIGURE 2:**
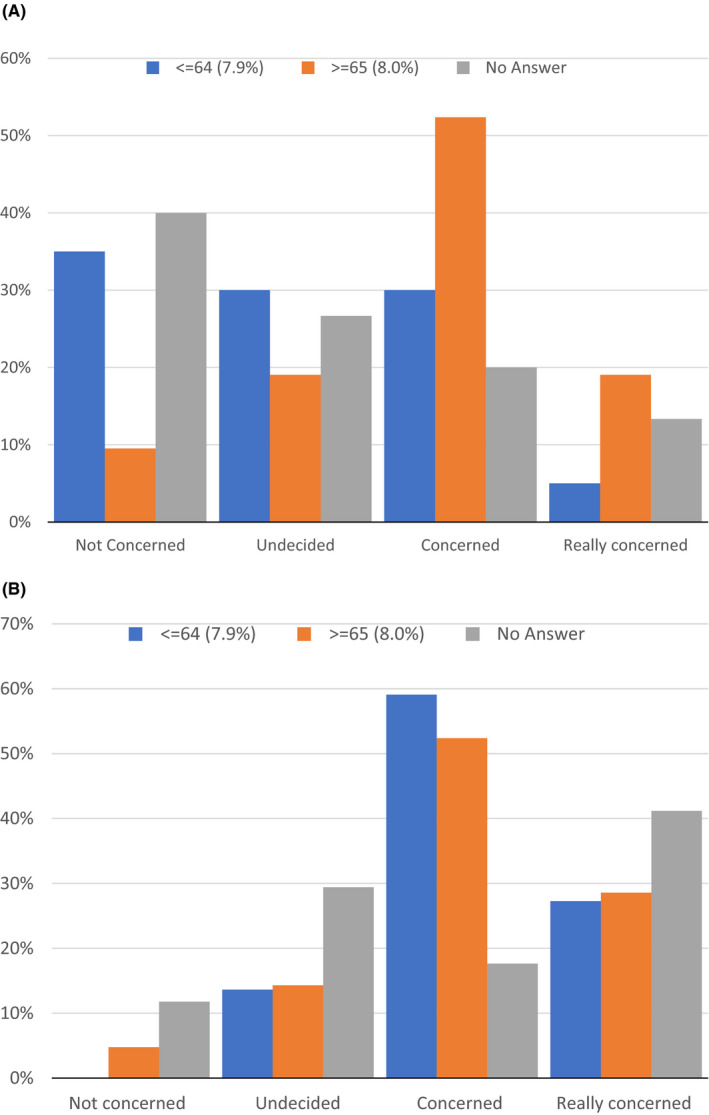
(A) What is your level of concern regarding your current blood glucose levels? Split by last reportedHbA1c mmol/mol. (B) How concerned are you by the possible longer term impact of having higher blood glucose levels? Split by last reported HbA1c mmol/mol

In relation to those with a last reported HbA1c of ≥65 mmol/mol, a majority of people (70%) were concerned or really concerned about the consequences of running a high HbA1c (Figure 2A). This contrasted with those who did not know their recent HbA1c, of whom only 33% were concerned or really concerned about the consequences of running a high HbA1c and those with an HbA1c ≤64 mmol/mol of whom only 35% were concerned about the shorter term consequences of high BG levels.

For longer term consequences in relation to HbA1c, for both for HbA1c ≤64 mmol/mol and ≥65 mmol/mol, there was concern in 85% and 80%, respectively, about their BG readings (Figure 2B). This was also true to a lesser extent in those who did not know their HbA1c (concern in 58% of those who did not know their recent HbA1c).

### 3.4 Patient views on and use of BG metres

**FIGURE 3:**
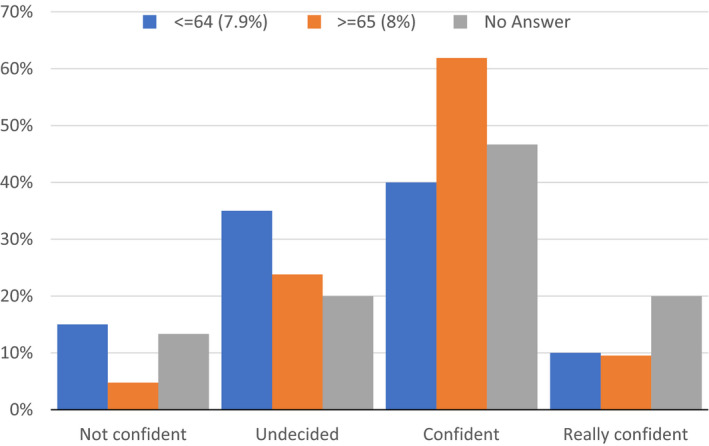
What is your level of confidence in your metre's accuracy? Split by last reported HbA1c (mmol/mol)

We next explored respondents' knowledge of BG metre performance in terms of accuracy and how this was reflected in their day‐to‐day practice. We found that 77% of respondents said that they had not ever discussed BG metre performance with a healthcare professional and 33% said that they had not been trained in use of their metre. Only 11% discussed performance of the BG metre with other people with T2DM. Only 21% regularly checked metre performance with a control solution and only 7% were aware of the International Standardisation Organisation (ISO) standards for BG metres.^6^ With regards to confidence about metre accuracy, this was generally high; 63% were confident in their metre's performance. For those with a HbA1c of ≤64 mmol/mol, 50% were confident or really confident about their metre's performance, with equivalent figures for those with a HbA1c of ≥65 mmol/mol being 72% and for those who did not know their HbA1c being 67% (Figure 3).

When questioned about adequacy of information about BG monitoring, 25% of individuals responded that they had sufficient information with 13% believing that the accuracy of their BG metre was being independently checked. Only 9% remembered discussing BG metre accuracy when their latest metre was provided.

Participants in our survey gave a wide variety of responses when asked what might reduce their concern. These provide us with insight into their day‐to‐day experience of BG monitoring and are listed in Appendix 2.

Examples included:

I do not expect these devices to be perfect but they should be reasonably correct.

I would like to have regular discussion about diabetes level and metre checking.

I don't know. The device was given to me by the hospital so have always presumed they were confident about the accuracy.

I have poor experience of professional advice because of stark changeover at my surgery.

Very little help is given regarding diet. As I have aged, now 75, I experience more Hypos.

As I am quite old (81 yrs), I don't feel the need to be anxious about how much life there is left in me! I have several 'chronic' conditions other than diabetes.

I need more education.

## 4 DISCUSSION

This study has shown that in a group of people with T2DM, concerns regarding long‐term complications were highly prevalent. This was seen in groups with both high and low levels of HbA1c, and if HbA1c was not known. This was in contrast to views of current BG levels where there was a noticeable lack of concern when HbA1c was not known. This is similar to our previous findings in people with T1DM.^7^, ^8^

We found significant concern about over‐ or under‐dosing of insulin. Only one‐fifth of patients responded that they had sufficient information. Only 9% remembered discussion of BG metre accuracy when their latest metre was provided. This indicates a large gap in patient education in this area, including about ISO standards.^6^ This is reflected in the fact that 30% of those questioned reported keeping their BG levels high to avoid hypoglycaemic episodes. These findings are of direct relevance to all involved in management of diabetes in a primary care setting, where increasingly T2DM individuals are exclusively managed.

Our findings provide important insights into the way that people view and react to their BG readings. This study would suggest that concern / anxiety about the longer term consequences of high BG levels is an effective motivator to encourage tighter glycaemic management. This was also described in an interview‐based qualitative study^10^ in people with diabetes undertaken to develop the Health and Self‐Management in Diabetes (HASMID^v1^) questionnaire. Some participants spoke of a worry of what diabetes and the implications of having diabetes would mean for them in the long‐term. This was linked to the level of understanding that they had on how diabetes could cause health problems in the future.

Responders to our survey reported lack of training on how to use the metres correctly (including the matter of quality control) and how to interpret and act on the data. Improvements in this area would have the biggest impact on diabetes self‐management and this would contribute to the debate on the role of BG monitoring in managing T2DM.

Respondents often did not know how accurate their metre was and did not have the opportunity to discuss metre performance with a health care professional when choosing a blood glucose metre. Patients reported little choice over the metre they are offered with local guidelines typically limiting the choice of equipment to control costs and ensure value for money. It's not unreasonable for patients to assume that the blood glucose monitoring equipment on offer/prescribed have been evaluated for effectiveness as well as cost ‐ so there is a degree of trust that what is being offered to them is of an adequate standard. This was apparent in some of the comments made by the respondents.

In the same study,^10^ some of those interviewed reported concerns over whether they were managing their BG levels correctly, achieving stable and consistent BG levels over a period of time, administering medication correctly, whether their diabetes was ‘stable’ and/or whether there had been any implications of having diabetes on areas of their health (such as neuropathy). Individuals noted that there was a degree of stress with the ‘review appointments’, and spoke of concerns of being ‘told off’ at these reviews.

In our study, we found that 52% of respondents were concerned that they might be over‐ or under‐dosing their insulin. Although 63% of people expressed confidence in the accuracy of their BG metre, only 25% of patients responded that they had sufficient information. In relation to the matter of BG metre accuracy when their latest metre was provided only 9% remembered a discussion about this. This indicates a large gap in patient education in this area. In some cases there may be limited attention paid to the accuracy/precision of the BG metres provided to patients as long as they fall within the fairly liberal ISO standards.^5^ This is reflected in the fact that 30% of those questioned reported keeping their BG levels high to avoid hypoglycaemic episodes.

In all, 51% of patients reported an HbA1c of 64 mmol/mol or less. That is lower than in our previous analysis of England general practice data^11^ but may reflect the fact that all the people studied here were taking insulin. Encouragingly, 75% reported having had an HbA1c check in the last 6 months. This is in keeping with our previous data,^12^, ^13^ although our survey was carried out before the coronavirus pandemic, which has resulted in many HbA1c tests not being performed on time in the UK,^14^ and probably elsewhere in the world according to a WHO survey describing disruption to diabetes services.^15^

Interestingly, we found that 77% of respondents said that they had not ever discussed BG metre performance and 33% said that they had not been trained in use of their metre. Furthermore, 31% had used the same BG metre for more than 3 years and only 44% recalled having been trained to use their BG metre. This suggests that there is scope for regular review of exactly what metre people are using and regular education updates on interpretation of BG metre readings and the importance of recording these, particularly given that only 42% of those questioned reported keeping a regular diary of BG readings day‐to‐day. Expert patient education programmes for people with T1DM such as DESMOND^16^ and X‐PERT^17^ can go a long way to provide the necessary information to alleviate anxiety about ‘balancing the equation’ for diet, exercise and insulin dosing and to build confidence in self‐management.

In an important outpatient questionnaire study from 2015, Ward et al^18^ described in a survey of people with T2DM that respondents’ most frequent personal pattern was to test “occasionally, as needed”, which did not differ by insulin use status, gender or age. Interestingly, in those people on insulin, significantly better control was found in those never experiencing being “too busy” than in those who were “too busy” several times per week. Similarly, never “feeling discouraged” as a barrier to self BG monitoring was associated with better metabolic control than experiencing the barrier a few times per week. Certainly this was borne out by some of the comments that patients provided to us such as:

Important especially if you are reacting to high or low reading or planning your meal, salad with low carbs. I take less insulin if reading is under 6 or more if having high carbs and reading is higher than 8.

It helps to understand how your diabetes is controlled. Plus when driving it is important for safety.

It needs to be correct.

Long as it’s not higher than 11 or lower than 5. Fine by me.

The group who was contacted for our survey comprised long‐term engaged people, but even within this group, there was significant variation in patient opinions, specifically; (a) awareness of the short‐term risks, (b) confidence in their ability to implement appropriate insulin dosage to adjust for shorter term variations in their daily life, (c) levels of awareness how to manage BG fluctuations and, (d) awareness of the limitations of BG monitoring technology. The figure of as many as 67% concerned about the long‐term impact of higher blood glucose levels is positive in terms of influence on medication concordance. In this regard Hashimoto et al^19^ reported that patients with T2DM, the patient's diabetes perception of ‘living an orderly life’ was associated with medication adherence.

We have previously shown that decisions taken in GP practices, including the decision to move to insulin treatment and provision of a BG metre have a profound influence on glycaemic outcomes in T2DM.^11^ Our analysis has shown both in modelling and in real world data that the effect of longer term multiple use of less precise BG monitoring strips resulted in an increase in longer term variability of actual BG levels in both models and as measured by HbA1c.^5^ We found that the increase in BG variability was over twice the change in variability in BG strips.

It should be pointed out that none of the people who replied to our survey were using a Flash blood glucose monitor.^20^, ^21^ This technology is increasingly being utilized by people with T1DM and in some with T2DM.^22^ Nevertheless, traditional BG monitoring will continue to be the way that most people who take insulin to treat diabetes monitor their diabetes for some time to come.

The study population was everyone who has signed up to ‘Research for the Future (RfTF)’.

We had no control of who responded to the survey which was presented online. However, tt is likely that many people with T2DM and on insulin were likely to reply to the survey, hence the high proportion of people with T2DM on insulin who responded to the survey.

### 4.1 Strengths and limitations

The survey used in this study was comprehensive and covered many of the aspects of day‐to‐day diabetes management and living with diabetes. Respondents were from across the spectrum of age and duration of T2DM.

Those contacted had already expressed an interest in participating in diabetes research and so there will be some degree of responder bias. Nevertheless the characteristics of responders were similar to those of non‐responders. The non‐responder rate was 62%. However this is not unusual for an online survey of this kind. There will be recall bias for the self‐reported HbA1c results reported.

We do not have specific information on demographic characteristics as this was an online survey. Ethnicity was not captured in our study as this is not generally self‐reported accurately.

The survey was online, so people who do not have access to a computer, tablet or smart phone were not able to respond. Furthermore, we have relied on self‐reported HbA1c and BG values. However Gonder‐Frederick et al^23^ reported that misrepresentation of test values by respondents was extremely rare. This is more likely to be true in well‐motivated groups such as those in our study.

## 5 CONCLUSION

In conclusion, there have been a large number of major developments in the treatment of T2DM in recent years. Feedback on service user experience is a powerful and clinically relevant tool to understand more clearly the strengths and weaknesses of current methods and services. The group who were contacted for this survey comprised long‐term engaged people but even within this group there was significant variation in patient opinions around (a) awareness of shorter term risks, (b) confidence in their ability to implement appropriate insulin dosage to adjust for shorter term variations in their daily life and, (c) awareness of the limitations of BG monitoring technology. Therefore, as in T1DM, there are areas where changes in education/support would benefit many people.

## CONFLICT OF INTEREST

No author has any conflict of interest.

## AUTHOR CONTRIBUTION

**Mike Stedman:** Conceptualization (equal); Formal analysis (lead); Investigation (equal); Methodology (equal); Project administration (equal); Visualization (lead); Writing‐review & editing (equal). **Rustam Rea:** Investigation (equal); Supervision (equal); Validation (equal); Visualization (equal); Writing‐original draft (equal); Writing‐review & editing (equal). **Christopher J. Duff:** Conceptualization (equal); Resources (equal); Visualization (equal); Writing‐review & editing (equal). **Mark Livingston:** Conceptualization (equal); Formal analysis (equal); Methodology (equal); Resources (equal); Software (equal); Writing‐original draft (equal); Writing‐review & editing (equal). **Katie McLoughlin:** Conceptualization (equal); Validation (equal); Visualization (equal); Writing‐original draft (equal); Writing‐review & editing (equal). **Louise Wong:** Methodology (equal); Visualization (equal); Writing‐original draft (equal); Writing‐review & editing (equal). **Stephen Brown:** Resources (equal); Software (equal); Validation (equal); Visualization (equal); Writing‐review & editing (equal). **Katherine Grady:** Methodology (equal); Project administration (equal); Resources (equal); Writing‐original draft (equal); Writing‐review & editing (equal). **Roger Gadsby:** Resources (equal); Software (equal); Supervision (equal); Validation (equal); Visualization (equal); Writing‐original draft (equal); Writing‐review & editing (equal). **John M. Gibson:** Conceptualization (equal); Resources (equal); Writing‐original draft (equal); Writing‐review & editing (equal). **Angela Paisley:** Investigation (equal); Writing‐review & editing (equal). **Anthony Fryer:** Formal analysis (equal); Validation (equal); Visualization (equal); Writing‐original draft (equal); Writing‐review & editing (equal). **Adrian H. Heald:** Conceptualization (lead); Formal analysis (supporting); Investigation (lead); Methodology (lead); Project administration (lead); Resources (lead); Supervision (lead); Validation (equal); Visualization (equal); Writing‐original draft (lead); Writing‐review & editing (lead).

## Supporting information

Figure S1

## Data Availability

The data that support the findings of this study are available from the corresponding author upon reasonable request.

## APPENDIX 1

### QUESTIONNAIRE

#### Question used (Responses= +Yes/No/Sometimes *Provided Scale Options ** Comment)

1. Age (years)*
2. Gender*
3. How long have you had Diabetes?*

#### Insulin use

4. On an average day, how many times do you inject insulin?*
5. On average how much insulin in total do you use each day?*
6. How much of the insulin you inject is basal / long acting insulin?*
7. For the more rapid acting insulin, how much on average do you inject each time?*
8. For the more rapid acting insulin, what amount would you adjust the amount each time you inject from one insulin injection to another?*
9. Do you use an insulin pump?+

#### Self blood glucose monitoring

10. Approximately how long have you been using Blood Glucose Meters and strips?*
11. On an average day how many times do you self‐check your blood glucose levels with strips & meter?*
12. How many different blood glucose meters do you currently use?*
13. Have you been trained to use any of these meters by a healthcare professional?+
14. Which types of meter are you currently using? Including Flash Glucose monitoring*
15. How long have you been using your latest meter?*

#### Results/actions

16. When calculating your insulin dose what Blood Glucose value do you use as target (Pre‐meal/Post‐meal/Bedtime)*
17. Do you keep a blood glucose diary?+
18. What blood glucose level do you see on average (Pre Meal/Post Meal/Bedtime)*
19. As an Estimate what is the highest BG level you have seen in the last week?*
20. As an Estimate What is the minimum Blood Glucose Level you have recorded in the last week?*
21. Do you ever keep your glucose levels higher because you are worried about having a hypo?+

#### Concern

22. How concerned are you by the possible longer‐term impact of having higher blood glucose levels?*
23. What was your last HbA1c reading?*
24. Approximately how long ago was your HBA1C done?*

#### Accuracy

25. What do you understand by the concept of accuracy with respect to Blood glucose?+*
26. Do you know and understand the current International Standardisation Organisation (ISO) standards as applied to BGM meters?+
27. When choosing your latest blood glucose meter, did your doctor/nurse discuss how accurate your meter is?+
28. How accurate do you think your current meter is within an accurate reading?*
29. Have you ever tested you meter with control solution?+
30. Do you regularly test your meter with control solution?+
31. Have you ever checked the accuracy of your meter by taking a second reading on your current meter?+
32. How much difference have you noticed between the first and second readings when testing the accuracy?*
33. Have you had to adjust your insulin dose as a consequence of any double check?+

#### Confidence

34. What is you level of concern regarding your current blood glucose levels?*
35. What is your level of confidence in your meter's accuracy?*
36. What might you do to eliminate or reduce this concern?**
37. Are you concerned that you might be over or under dosing your insulin?+

#### Support

38. Have you ever discussed an issue of accuracy with your health professional?+
39. Have you discussed/talked about this issue of accuracy with other patients with diabetes?+
40. Do you feel there is adequate information and support available to you about your Blood Glucose Monitoring meter?+
41. Do you believe that the accuracy of your meter is being independently checked?+
42. Do you believe that the NHS should carry out its own independent checks on meter accuracy?+
43. Is there anything else you would like to tell us?**

## APPENDIX 2

### RESPONSES TO THE OPEN QUESTION ‘IS THERE ANYTHING MORE YOU WOULD LIKE TO TELL US.’

#### Accuracy

- *I do not expect these devices to be perfect but they should be reasonably correct*.
- *I feel that any reading is at that moment. When I have a reading of 6 and minutes later 4, that worries me*.
- *I think this refers to how close the reading is to the actual value*.
- *I would expect the meter to be accurate*.
- *Only that insulin take is dependent on the reading. Wrong reading =* *wrong dosage*.
- *Readings have a tolerance between 1–2%*.
- *Had never thought of checking meter with control solution more frequently ‐ mine is out of date ‐ so thank you. I know it is in the literature but you don't read this every time you get a new cassette so may not pick up differences listed by the manufacturer*.
- *I am confident with the accuracy of my accucheck meter but confirmation about accuracy from a health professional would be helpful*.
- *I am told that each person is different as to effect of any particular insulin dose. so my take on meter accuracy is how effective my subsequent insulin dose has been by taking a post dose reading*.

#### Why it is important to control BG levels

- *If you don't control your blood sugars you can get more health issues*.
- *Managing your health probably*.
- *So you can keep your levels low because of risk of stroke etc*.
- *That accuracy is important to avoid hypo or hyper*.
- *To keep below 7 mmol if possible*.
- *I do have a glycogen storage disease, type 5, so it is possible that this could be causing problems with my glucose levels. However, in spite of all the health professionals I have discussed this with agreeing that it may complicate glucose control my diabetic consultant disagrees. My glucose levels can suddenly drop without any change to my medication, eating or lifestyle*.
- *I would like to have regular discussion about diabetes level and meter checking*.

#### Concerns

- *As my average meter readings are confirmed by my HbA1c, I am happy to rely on my meter ‐ if I was concerned I would change it*.
- *Carry out more comparisons and/or consult my GP*.
- *Change meter/control my diet better with more exercise*.
- *Check BG more often*.
- *Check bloods at clinic*.
- *Check glucose levels more than twice a day*.
- *Check its accuracy*.
- *Check with a control solution*.
- *Constant vigilance regarding what I eat and the amount of exercise I do*.
- *Does the meter really recall correct sugar reading?*
- *Double testing is important*.
- *More training for patients on how to use the meter regarding how to understand results*.
- *Find out of I should have been given control solution*.
- *Get my meter callibrated against a control solution*.
- *Have better professionally advice than I currently have*.
- *HAVE MY METER CHECKED ON MY ANNUAL CHECK UP*.
- *I don't know. The device was given to me by the hospital so have always presumed they were confident about the accuracy*.
- *I would ask my nurse specialist at my next appointment*.
- *Keep on eye on my diet*.
- *More professional monitoring and information*.
- *No choice in meter selection as decided by the general practice who will only supply test strips on a prescription meter. Meter not as good as AVIVA meter I had in the past although I like the Bluetooth to my phone with my AgaMatrix*.
- *Reduce current stress*.
- *Talk to diabetes nurse, who checks records and suggests new doses.......changes meter occasionally*.
- *To look at how the results affect my diabetes. If results were different ask diabetes nurse to check my glucose levels again*.
- *Need more education*.
- *I think that the NHS should have seminars or meeting's with patients to discuss there concerns or issues*.

#### Self perception

- *I have been a diabetic for more than 50 years my body tells me if things are not as they should be I trust it*.
- *I feel confident that with modern technology my meter is accurate to the level I need*.
- *I have been prescribed insulin and gliclazide but have recently been told to stop taking gliclazide; since then my glucose readings have almost always been in range*.
- *I have poor experience of professional advice because of stark changeover at my surgery*.
- *I need more education*.
- *I personally use the DAFNE, dose adjustment for normal eating, and it works very well for me*.
- *Before this regime my blood glucose levels were all over the place. My diabetes health professionals are very good at monitoring me and in advising me of changes*.
- *I think taking responsibility for one's diabetes and doing the tests and making the adjustments is my problem*.
- *I was diagnosed with type 2 diabetes, 30 yrs ago. Quite mild at first, controlled by diet. I later went on medication, glipizide Metformin. This continued until approximately 2 yrs ago when it was decided that I needed to go on insulin. I enjoy very good health for my age. I take plenty of exercise and eat healthily, so I am quite happy with my regimen. Hope this information is useful to you*.

#### Overall

- *Preferably be issued with a patch that can be swiped e.g. a free style libre. But they are only issued to type 1s*.
- *My aim is too keep below 7 mmol if possible. My blood meter was supplied by the NHS, no discussion took place*.
- *Trying to be consistent in a busy lifestyle and avoiding hypos or hypers*.
- *If you are new to insulin as I am and trying to find the right dose then this is pretty hard even when I am a health professional myself. The support of my nurse has been critical and excellent. I think I assumed that the meters would be sufficiently calibrated and accurate but the whole process of pricking fingers seems a bit old‐fashioned if there are better and more accurate devices to track blood sugar*.
- *It is vital to keep my blood glucose down because if not then I will be more likely to develop Diabetic complications*.
- *We need to keep it (blood glucose) low to avoid nerve damage and other health problems*.
- *Why does the NHS not standardize the type of meter given out?*
- *Publishing the results of any research into the accuracy of blood glucose monitors would help users chose the best device and would be really helpful. My own monitor was provided free of charge by ACCU‐CHECK. I would be happy to purchase a superior monitor if need be*.
- *The only check/verification of my regular daily blood sugar readings is the HbA1c which in my case only gets done every 3 months. I do not think that check is done often enough*.
- *This is the device that allows me to be confident that I am managing my blood glucose levels adequately. If these devices are not seen as reliable why are we wasting all this money?*
- *It would be marvellous if finger pricking could be stopped as a routine method of blood reading, needle changing and disposal is quite embarrassing in public. Finding a public toilet to read/test is sometimes quite difficult as are car parks*.
- *Very little help is given regarding diet. As I have aged, now 75, I experience more Hypos*.
- *A diabetic should not pay for the meter. We want to be healthy, it should be given to us free ‐ and also to be updated every 2 years or so*.
- *As I am quite old (81 yrs), I don't feel the need to be anxious about how much life there is left in me! I have several ‘chronic’ conditions other than diabetes. They all seem to be managed satisfactorily these days, and I enjoy a rich quality of life*.
