# Supplementary material for: The experience of blood glucose monitoring in people with type 2 diabetes mellitus (T2DM)
Source: Endocrinol Diabetes Metab. 2021 Dec 17;5(2):e00302. doi: 10.1002/edm2.302 (PMC8917860; doi:10.1002/edm2.302)
Supplement: Supplementary file 1 — Figure S1 [file EDM2-5-e00302-s001.docx]

**Supplementary Figure: Are you concerned that you might be over or under dosing your insulin? Split by last reporded HbA1c (mmol/mol)**
